# Influence of Human Papillomavirus Infection on the Natural History of Cervical Intraepithelial Neoplasia 1: A Meta-Analysis

**DOI:** 10.1155/2017/8971059

**Published:** 2017-07-24

**Authors:** Mingzhu Liu, Xiaolong Yan, Mei Zhang, Xiaoju Li, Shugang Li, Mingxia Jing

**Affiliations:** ^1^Department of Public Health, Shihezi University School of Medicine, Shihezi, Xinjiang, China; ^2^The Key Laboratories for Xinjiang Endemic and Ethnic Diseases, Shihezi University, Shihezi, Xinjiang, China

## Abstract

**Objective:**

To provide a scientific basis for the prevention and treatment of cervical intraepithelial neoplasia grade 1 (CIN1). This study evaluated the impact of human papillomavirus (HPV) infection on the natural history of CIN1.

**Methods:**

Electronic databases of Cochrane Library, EMBASE, PubMed, CNKI, CBM, and Wanfang were searched in April 2016. The eligibility criteria were documented by Preferred Reporting Items for Systematic Reviews and Meta-Analyses (PRISMA). We used the Newcastle-Ottawa scale (NOS) to assess study quality.

**Results:**

Thirty-eight studies out of 3,246 identified papers were eligible for inclusion. The risk of CIN1 progression (relative risk [RR]: 3.04; 95% confidence interval [CI]: 2.41–3.83; *P* < 0.00001) and persistence (RR: 1.48; 95% CI: 1.17–1.87; *P* = 0.001) was higher in the HPV-positive group than HPV-negative group. Specifically, the risk of CIN1 progression (RR: 13.91; 95% CI: 3.46–55.90; *P* = 0.000) was higher among persistent high-risk HPV-positive patients and the ratio of CIN1 regression (RR: 0.65; 95% CI: 0.59–0.71; *P* < 0.00001) was lower in the HPV-positive group than HPV-negative group.

**Conclusion:**

HPV infection resulted in an increased risk of CIN1 progression and decreased disease reversibility. Persistent high-risk HPV infection resulted in a further increased risk of CIN1 progression.

## 1. Introduction

Cervical intraepithelial neoplasia grade 1 (CIN1) is a precancerous lesion closely related to cervical cancer and characterized by a shorter and less observable clinical course. There is no consensus for intervention and treatment of CIN1, and there are currently no clear markers to predict disease progression and regression [1]. Human papillomavirus (HPV) is a major causative pathogen of reproductive tract infections and can induce the immortalization of normal cells, which precedes their malignant transformation. Approximately 90% of CIN cases and over 99% of cervical cancer cases occur in HPV-positive patients [2]; therefore, HPV testing has become a major component of cervical disease screening, diagnosis, and follow-up. The association between CIN1 and HPV remains controversial. The results of several studies suggest that CIN1 is mainly caused by low-risk HPV infection [3–5]. However, there is also evidence that high-risk HPV is strongly associated with CIN1 [6, 7]. Differences in the risk of HPV infection and CIN1 disease outcome [8, 9] may be due to regional differences in populations. Furthermore, there are scarce independent systematic reviews on the effects of HPV infection and CIN1. This study evaluated the impact of HPV infection on the natural history of CIN1 by conducting a literature review in order to provide a scientific basis for the prevention and treatment of CIN1.

## 2. Methods

### 2.1. Electronic Literature Databases

A systematic search was conducted using the Cochrane Library, Excerpta Medica database (EMBASE), PubMed, China National Knowledge Infrastructure (CNKI), Chinese Biomedical Literature Database (CBM), and the Wan fang Data. The literature search was performed on April 20, 2016. The PICOS items were identified (see Appendix 1 in Supplementary Material available online at https://doi.org/10.1155/2017/8971059) in this study as follows: P, cervical intraepithelial neoplasia grade 1 (CIN1); I, HPV positivity; C, HPV negative; O, the relative risk (RR) of progression, persistence, and regression of CIN1 in HPV-positive and HPV-negative patients being compared; S, retrospective studies and prospective studies. The search strategies were determined (the specific search strategy is described in Appendix 2) before the study. The MESH search terms for PubMed included the following: (“Squamous Intraepithelial Lesions of the Cervix”[MeSH] OR low-grade squamous intraepithelial lesion OR mild cervical dysplasia OR CIN1 OR mild Cervical Intraepithelial Neoplasia) AND (“Human Papillomavirus DNA Tests”[MeSH] OR human papillomavirus detected OR human papillomavirus test OR human papillomavirus infection) AND (Cohort Study OR follow up).

### 2.2. Inclusion and Exclusion Criteria

We systematically reviewed published studies according to the following inclusion criteria: studies examining the impact of HPV infection on the natural history of CIN1 disease; studies including at least HPV-negative and HPV-positive; results at the start and end of follow-up including cervical histology or cytology, a diagnosis consistent with the CIN classification system or atypical hyperplasia (dysplasia) and the carcinoma in situ (CIS) classification system; patients diagnosed with CIN1 who did not undergo interventions including cryosurgery, electrocoagulation therapy, laser therapy, microwave therapy, cold knife conization, loop electrosurgical excision procedure, and trachelectomy; follow-up observation for at least 6 months; complete information so that each document contained sufficient information to calculate statistical indicators of relative risk (RR) or 95% confidence intervals (CIs). The exclusion criteria were as follows: studies that did not meet the inclusion criteria, literature reviews, the absence of a control group, and duplicate publications. We also excluded papers with incomplete initial data.

### 2.3. Quality Assessment

The Newcastle-Ottawa scale (NOS), recognized as a good study quality assessment tool, was used to assess the quality of the studies identified in our literature search (see Appendix 3). The evaluation system included eight literature evaluation entries for a total of nine possible points [44], including the selection of the study population, comparability, exposure assessment, and the results of the evaluation. The NOS scale validity rating criteria are as follows: 8-9, high quality; 6-7, medium quality; <5, low quality.

### 2.4. Data Collection

Two authors (Mingzhu Liu and Xiaolong Yan) independently extracted data and crosschecked their data after aggregating the results. Disagreements were resolved by discussion with Professor Mingxia Jing. Data were collected at the start of the study, including basic information, background and characteristics of the research object, and disease diagnosis and evolution. This information is presented in Table 1.

### 2.5. Data Analysis

Thirty-eight articles were analyzed using RevMan 5.0 (Cochrane systems IMS) and Stata 12.0 (Stata Corp, College Station, Texas, TX, USA). To assess the heterogeneity among studies, we calculated the *I*^2^ index. Low and high levels of heterogeneity were considered as *I*^2^ ≤ 50% and >50%, respectively. We use a fixed model on the conditions of *P* > 0.05 and *I*^2^ ≤ 50%. We use a randomized model on the conditions of *P* < 0.05 or *I*^2^ > 50%. Combined effects were estimated as relative risk (RR) values with 95% confidence intervals (CIs). All reported *P* values were two-sided, and a significance level of 0.05 was used. Subgroup analyses were also performed by HPV type (high-risk HPV and low-risk HPV), study design (retrospective and prospective studies), regional population distribution (Asian, European, and American populations), sample size (<100 cases, 100–500 cases, and >500 cases), and follow-up time (6–18 months, 18–24 months, and >24 months). Sensitivity analyses were performed using Stata 12.0.

## 3. Results

### 3.1. Search Result

**Figure 1:**
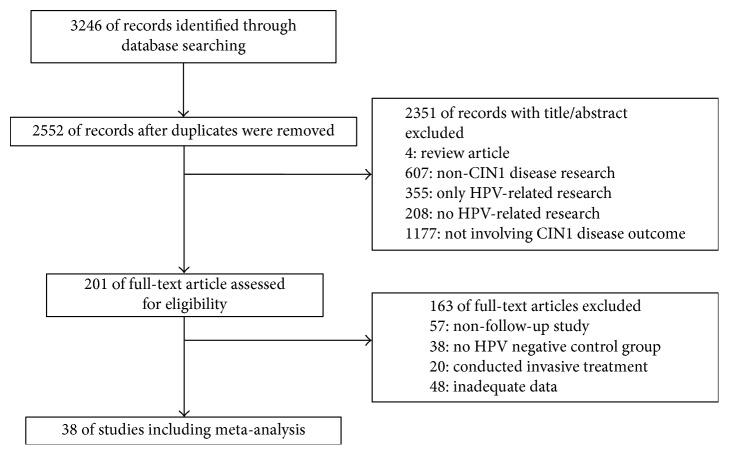
Flowchart of identifying and including studies.

Figure 1 shows the study selection process. Initially, 3,246 articles were included in our search strategy. A total of 38 articles, including 9,758 patient cases, were finally included in the analysis, based on the inclusion and exclusion criteria. A total of 27 studies assessed regression of CIN1 to a normal status. Twenty-five studies examined persistent CIN1 and 36 articles evaluated the progression from CIN1 to high-grade cervical intraepithelial neoplasia and cervical cancer (CIN2+).

### 3.2. Basic Characteristics and Quality Assessment of the Included Studies

The basic features of the 38 studies included in this meta-analysis are listed in Table 1. The studies spanned a period of 30 years (1986 to 2016) and included 22 prospective and 16 retrospective studies. The study sample sizes ranged from 29 [41] to 2,009 [30] cases. Twenty-seven studies reported median/mean ages, ranging from 16 to 76 years. The mean follow-up time ranged from 6 to 96 months. The studies included Asian (16 studies), European (14 studies), and American (8 studies) populations. Thirty-eight studies had quality ratings between 7 and 9 points, with an average of 7.84 points. Twenty-eight articles had quality scores ≥8 points, corresponding to high-quality research, while 10 articles were of medium quality.

## 4. Results of the Meta-Analysis

### 4.1. The Influence of HPV Infection on the Outcome of CIN1 Lesions

**Figure 2:**
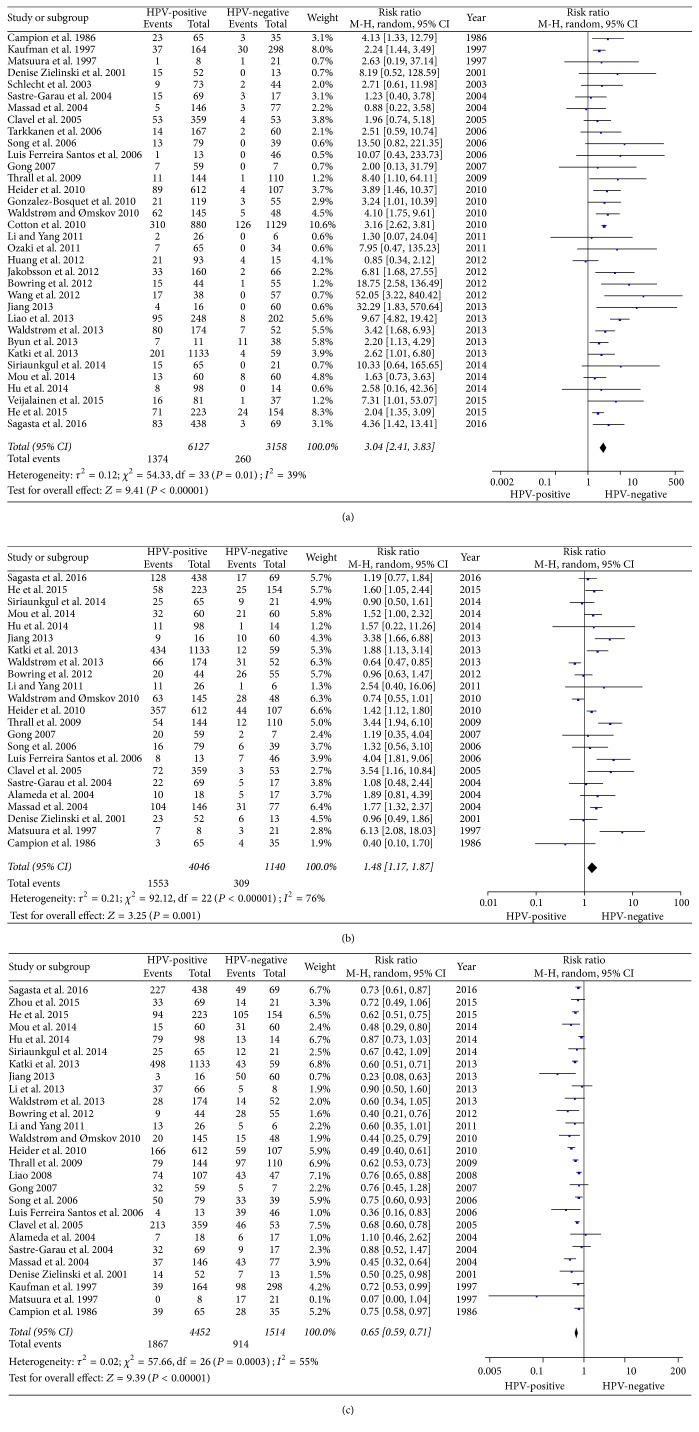
Forest plot of HPV-positive patients and CIN1 disease outcomes. HPV positivity in the exposed group and HPV negativity in the control group. (a) Forest plot of HPV positivity and CIN1 disease progression; (b) Forest plot of HPV positivity and CIN1 disease persistence; (c) Forest plot of HPV positivity and CIN1 disease regression.

A total of 38 studies were included in this study, and of these, 34 estimated the impact of HPV infection on the progression of CIN1 lesions; pooled analysis showed that the risk of CIN1 disease progression was 3.04-fold higher in the HPV-positive group than in the HPV-negative group (95% CI: 2.41–3.83; *Z* = 6.28; *P* < 0.00001), with low heterogeneity (*P* = 0.01; *I*^2^ = 39%; Figure 2(a)). Twenty-three studies estimated the impact of HPV infection on the persistence of CIN1 lesions; pooled analysis showed that the risk of CIN1 disease persistence was 1.48-fold higher in the HPV-positive group than in the HPV-negative group (95% CI: 1.17–1.87; *Z* = 3.25; *P* = 0.001), with significant heterogeneity (*P* < 0.00001; *I*^2^ = 76%; Figure 2(b)). Twenty-seven studies estimated the impact of HPV infection on the regression of CIN1 lesions; pooled analysis showed that the ratio of CIN1 disease regression was 0.65-fold lower in the HPV-positive group than in the HPV-negative group (95% CI: 0.59–0.71; *Z* = 9.39; *P* < 0.00001), with high heterogeneity (*P* = 0.0003; *I*^2^ = 55%; Figure 2(c)).

In subgroup analyses, the risk of the persistence of CIN1 was higher in American than European or Asian populations (RR_Asian_ = 1.54, 95% CI: 1.17–2.02; RR_European_ = 0.97, 95%CI: 0.73–1.30; RR_American_ = 2.29, 95%CI: 1.59–3.28; *P* = 0.001). The ratio of regression of CIN1 was higher in patients followed up for 18–24 months than in those followed up for 6–18 months or >24 months (RR_6–18_ = 0.61, 95% CI: 0.53–0.70; RR_18–24_ = 0.73, 95% CI: 0.67–0.80; RR_>24_ = 0.55, 95% CI: 0.46–0.66; *P* = 0.007). Significant differences in HPV type, study design, and sample size were not detected (see Appendix 4).

### 4.2. The Influence of HR-HPV Infection Time on CIN1 Lesions

Long or short HR-HPV infection times had different effects on CIN1 lesion history. Persistent HR-HPV infection means that, in two or more times, the HR-HPV detected was positive and transient HR-HPV infection means that, in only one time, the HR-HPV detected was positive [6]. The risk of CIN1 disease progression was 13.91-fold higher in the persistent HR-HPV infection group than in the HPV-negative group (95% CI: 3.46–55.90; *P* = 0.000); the ratio of CIN1 disease regression was 0.61-fold lower in the persistent HR-HPV infection group than in the HPV-negative group (95% CI: 0.47–0.80; *P* = 0.000). The impact of transient HR-HPV infection on CIN1 disease progression and regression was not statistically significant. Furthermore, persistent and transient HPV infection did not have a significant impact on CIN1 persistence (Table 2).

### 4.3. Sensitivity Analysis

We conducted a sensitivity analysis for the progression, persistence, and regression of CIN1 disease, respectively (see Appendix 5). All of the included studies were distributed evenly from the central line, with no significant deviation. Therefore, no individual study affected the pooled effect results.

## 5. Discussion

A total of 38 studies were included in the current study. Of these, 23 studies examined HR-HPV infections and three of these also considered LR-HPV infection. Three studies assessed HPV infection times. Studies have shown that HPV infections are associated with an extended disease course in CIN1, increasing the risk of disease progression and hampering the reversal of CIN1. Persistent HR-HPV infection was a major factor associated with CIN1 progression. This finding provides important data for the clinical management of CIN1 disease, in order to avoid excessive or inadequate treatment. Regional population distribution and follow-up time were also associated with CIN1 disease outcome.

In the HPV-positive group, the risks of CIN1 progression, persistence, and regression, respectively, were 3.02, 1.45, and 0.65, compared to the HPV-negative group. A randomized controlled study from the atypical squamous cells of undetermined significance-low-grade squamous intraepithelial lesion (ASCUS-LSIL) (ALTS) group [45] reported higher risks of CIN1 progression and persistence (12.34 and 2.41, resp.) in the HPV-positive group than that observed in our study. The ratio of CIN1 regression in the HPV-positive group was 0.19, which is lower than that found in this study. These findings may be explained by the fact that 34.2% of the included studies involved population-based screening and identification of patients with CIN1 was the object of study. Compared to the ALTS study, with subjects from four large clinical centers, the patient's condition is relatively severe. However, this study reached a comparable conclusion. HPV positivity was associated with hampered CIN1 lesion regression and increased risk of disease progression and persistence.

Our research on the impact of HPV type and infection duration on CIN1 disease history found that persistent HR-HPV infection was a major risk factor for CIN1 progression to CIN2+, while LR-HPV and transient infections were not significantly associated with increased risk of CIN1 progression. The results are corroborated in a large prospective study by Dalstein et al. [46]. Furthermore, only Huang et al. [23] explored the relationship between human papillomavirus type 16 (HPV16), HPV18, and other oncogenic HPV and CIN1 disease, reporting that the risk of CIN1 progression to CIN2/3 in patients with HPV16 infection was 2.51 and 6.95 times that in patients with HPV18 infection and other oncogenic forms, respectively. HPV16 is considered the major risk factor for CIN1 disease progression.

Regional population distribution and follow-up time were the two main factors influencing CIN1 disease history. The risk of disease progression in HPV-positive CIN1 patients in the Americas was lower than that of patients in Asian and European countries (RR_Asian_ = 3.94; RR_European_ = 3.10; RR_American_ = 2.31; *P* = 0.380), and the ratios of disease regression (RR_Asian_ = 1.54; RR_European_ = 0.97; RR_American_ = 2.29; *P* = 0.001) and persistence (RR_Asian_ = 0.70; RR_European_ = 0.69; RR_American_ = 0.56; *P* = 0.060) were higher than those in Asia and European countries. In these Americas countries, 87.5% are North Americas countries (seven US and one Brazilian states). Previous studies have shown that a screening strategy of ThinPrep cytologic tests (TCTs) combined with HPV tests has gained popularity [43]. CIN1 had a lower probability of progression to invasive cervical cancer (ICC) and a higher probability of regression. In some Asian and European countries, screening is performed mainly by visual inspection with acetic acid or iodine and Pap smears. These methods are less costly than TCT and HPV DNA tests [47]. Therefore, the probability of CIN1 progression was relatively high and the probability of disease reversal was relatively low. Numerous studies have shown gradual CIN1 regression with clearance of HPV infections, and our study found that natural clearance of HPV infections may take longer than 24 months. This is consistent with the interval of two years or longer for HPV-based cervical cancer screening [48–50].

This study has the following advantages. First, the well-designed studies provided strong evidence for the analysis of the influence of HPV infection on CIN1 disease history. Furthermore, the full search was relatively comprehensive and included a large number of studies, significantly increasing the sample size compared with using the single original research study criteria; therefore, the combined effect size was more accurate. Second, subgroup analyses were performed according to HPV type, study design, regional population distribution, sample size, and follow-up time, in order to explore potential confounders. Some limitations should be considered when interpreting the results of this study. First, CIN1 is affected by many factors and we were unable to control for parameters such as age at first sexual intercourse, number of pregnancies and delivery times, and individual immune status. Second, most studies included a larger age range; therefore, we did not conduct subgroup analysis by patient age. Third, more papers from China were included in the present study, which would limit the significance. This conclusion needs to be further testified in the people from other countries. Finally, there are few studies on low-risk HPV infection potentially affecting the results of subgroup analysis of HPV type. Therefore, we will research low-risk HPV infection in future studies to improve the accuracy of these results.

## 6. Conclusion

HPV infection resulted in an increased risk of CIN1 progression and reducing disease reversibility. Persistent high-risk HPV infection resulted in a further increased risk of CIN1 progression. Furthermore, regional population distribution and follow-up times influenced CIN1 disease history.

## Supplementary Material

Appendix 1: The PICOS Principles in the PRISMA Statement. Appendix 2: Full Search strategy. Appendix 3: Newcastle-Ottawa Quality Assessment Scale: Cohort Studied. Appendix 4: Subgroup Analyses. Appendix 5: Sensitivity Analysis.

## Figures and Tables

**Table 1:** Basic characteristics and quality assessment of included studies.

Study	Design	Age (years)	Region (subgroup)	HPV type	HPV infection time	Indexes	Follow-up time (months)	Quality rating
Sagasta et al. (2016) [10]	Prospective	33 ± 10	Spain (Europe)	HR-HPV	NR	①②③	28	8
Veijalainen et al. (2015) [11]	Retrospective	40.4	Finland (Europe)	HR-HPV	NR	①	96	8
He et al. (2015) [12]	Retrospective	35 ± 16.93	China (Asia)	HR-HPV	NR	①②③	15 (8–24)	9
Zhou et al. (2015) [13]	Prospective	37.57 ± 9.12	China (Asia)	HR-HPV	NR	③	24	7
Mou et al. (2014) [14]	Retrospective	38.18 ± 4.26	China (Asia)	NR	NR	①②③	36	7
Siriaunkgul et al. (2014) [15]	Prospective	46.6	Thailand (Asia)	NR	NR	①②③	24	8
Hu et al. (2014) [16]	Prospective	30–59	China (Asia)	HR-HPV	Transient	①②③	24	9
					Persistent	①②③		
Jiang (2013) [17]	Retrospective	39.16 ± 8.97	China (Asia)	HR-HPV	NR	①②③	24	8
Waldstrøm et al. (2013) [18]	Prospective	32.3	Denmark (Europe)	NR	NR	①②③	60	8
Katki et al. (2013) [19]	Prospective	30–64	United States (America)	NR	NR	①②③	60	8
Byun et al. (2013) [20]	Prospective	46	Korea (Asia)	HR-HPV	NR	①	8	8
Liao et al. (2013) [21]	Prospective	30–49	China (Asia)	HR-HPV	NR	①	36	8
Li et al. (2013) [22]	Prospective	38	China (Asia)	HR-HPV	NR	③	6	8
Wang et al. (2012) [6]	Retrospective	35.4 (20–53)	China (Asia)	HR-HPV	Persistent	①	18.6 (8–24)	9
Huang et al. (2012) [23]	Retrospective	30 (22–70)	China (Asia)	HR-HPV	NR	①	24	7
Bowring et al. (2012) [24]	Prospective	36.8 ± 10.2	Britain (Europe)	HR-HPV	NR	①②③	12	8
Jakobsson et al. (2012) [25]	Retrospective	34	Finland (Europe)	HR-HPV	NR	①	6	8
Ozaki et al. (2011) [26]	Prospective	39	Japan (Asia)	NR	NR	①	17	7
Li and Yang (2011) [5]	Prospective	30 ± 2.32	China (Asia)	HR-HPV	NR	①②③	6	8
				LR-HPV		②③		
Gonzalez-Bosquet et al. (2010) [27]	Prospective	32.25	Germany (Europe)	HR-HPV	NR	①	25	7
Waldstrøm and Ømskov (2010) [28]	Retrospective	32	Denmark (Europe)	NR	NR	①②③	36	8
Heider et al. (2010) [29]	Retrospective	33	United States (America)	HR-HPV	NR	①②③	34	9
Cotton et al. (2010) [30]	Prospective	20–59	Britain (Europe)	HR-HPV	NR	①	36	8
Thrall et al. (2009) [7]	Prospective	≥30	United States (America)	HR-HPV	NR	①②③	24	9
Liao (2008) [31]	Prospective	30–49	China (Asia)	HR-HPV	NR	③	24	7
Gong (2007) [1]	Prospective	38.37 ± 5.26	China (Asia)	HR-HPV	Transient	①②③	24	7
					Persistent	①②③		
Luis Ferreira Santos et al. (2006) [32]	Prospective	31 (16–63)	United States (America)	NR	NR	①②③	12	8
Tarkkanen et al. (2006) [33]	Prospective	35 (20–60)	Finland (Europe)	NR	NR	①	6	7
Song et al. (2006) [34]	Retrospective	38	Korea (Asia)	NR	NR	①②③	24	8
Clavel et al. (2005) [35]	Retrospective	30	France (Europe)	HR-HPV	NR	①②③	24	8
Massad et al. (2004) [36]	Prospective	37.4	United States (America)	HR-HPV	NR	①②③	90	8
				LR-HPV	NR	①②③		
Sastre-Garau et al. (2004) [37]	Retrospective	31	France (Europe)	HR-HPV	NR	①③	24	8
Alameda et al. (2004) [38]	Retrospective	25–45	Spain (Europe)	HPV	NR	②③	24	7
Schlecht et al. (2003) [39]	Retrospective	16–65	Brazil (America)	HR-HPV	NR	①	53.3	8
				LR-HPV	NR	①		
Denise Zielinski et al. (2001) [40]	Retrospective	40.5 (20–76)	Holland (Europe)	HR-HPV	NR	①②③	16.8 (0–54)	8
Matsuura et al. (1997) [41]	Prospective	NR	United States (America)	NR	NR	①②③	89.2 ± 25.2	8
Kaufman et al. (1997) [42]	Retrospective	NR	United States (America)	HR-HPV	NR	①③	6	7
Campion et al. (1986) [43]	Prospective	<30	Britain (Europe)	NR	NR	①②③	22.4 (19–30)	7

*Note*. ①: the relative risk (RR) of progression of CIN1 patients of HPV-positive compared with the HPV-negative; ②: the relative risk (RR) of persistence of CIN1 patients of HPV-positive compared with the HPV-negative; ③: the relative risk (RR) of regression of CIN1 patients of HPV-positive compared with the HPV-negative. HPV, human papillomavirus; HR-HPV, high-risk HPV; LR-HPV, low-risk HPV; HPV(+), HPV-positive; NR, not reported.

**Table 2:** The influence of HR-HPV infection time on CIN1 lesions.

Disease outcome	Infection time	Number of studies	Heterogeneity test results			Merged effect RR value (95% CI)	*P* value
			*Q* Value	*P* Value	*I* ^2^(%)		
Progression	Persistent	3	2.49	0.290	20	13.91 (3.46, 55.90)	0.000
	Transient	2	0.05	0.820	0	1.06 (0.12, 9.01)	0.960
Persistent	Persistent	2	0.22	0.640	0	2.15 (0.75, 6.18)	0.160
	Transient	2	0.02	0.890	0	0.57 (0.17, 1.92)	0.360
Regression	Persistent	2	1.86	0.170	46	0.61 (0.47, 0.80)	0.000
	Transient	2	0.22	0.640	0	1.03 (0.86, 1.24)	0.750

RR, relative risk; CI, confidence interval.
