# Supplementary material for: Influence of Human Papillomavirus Infection on the Natural History of Cervical Intraepithelial Neoplasia 1: A Meta-Analysis
Source: Biomed Res Int. 2017 Jul 24;2017:8971059. doi: 10.1155/2017/8971059 (PMC5546131; doi:10.1155/2017/8971059)
Supplement: Supplementary file 1 — Appendix 1: The PICOS Principles in the PRISMA Statement. Appendix 2: Full Search strategy. Appendix 3: Newcastle-Ottawa Quality Assessment Scale: Cohort Studied. Appendix 4: Subgroup Analyses. Appendix 5: Sensitivity Analysis. [file 8971059.f1.docx]

SUPPLEMENTARY APPENDIX

Accompanying the manuscript:

**Influence of Human Papillomavirus Infection on the Natural Outcome of Cervical Intraepithelial Neoplasia 1 : A Meta-Analysis**

Mingzhu Liu

Xiaolong Yan

Mei Zhang

Xiaoju Li

Shugang Li, Associate professor, M.D., Ph.D.

Mingxia Jing , Professor, M.D., Ph.D.

Appendix 1:The PICOS Principles in the PRISMA Statement

Appendix 2: Full Search strategy

Appendix 3. Newcastle-Ottawa Quality Assessment Scale: Cohort Studied

Appendix 4. Subgroup Analyses

Appendix 5. Sensitivity Analysis

**Appendix 1:The PICOS Principles in the PRISMA Statement**

| **Description** | **Explanation** | **Specific Content** |
| --- | --- | --- |
| P  The subjects or defined diseases | Provides a set of population information (disease information and context factors) that need to be defined in relation to the participant (usually the patient). | Study of Disease  Cervical mild atypical hyperplasia (CIN1) that atypical cells confined to the lower third of the epithelium, according to the international standard out of the histological diagnosis. |
|  |  | Demographic Factors  The age is 15 years old or above;Region including Asia (mainly China, Japan, Korea and North Korea, etc.), Europe and the United States (mainly the United States, Germany, Britain, Italy, etc.); Source of population. |
|  |  | Important Features  All patients were only followed up and were not fully treated (including cryotherapy, electrocoagulation therapy, laser therapy, microwave therapy, cold knife cervical conization, cervical electrosurgery and cervical resection ). |
| I  Intervention /Exposure | The interventions (exposures) involved in the systematic review / meta-analysis should be clearly reported. Other interventions (exposures) may include diagnosis, prevention or treatment, specific treatment process arrangements, lifestyle changes, psychological or educational interventions, or risk factors. | Exposure factors were HPV infection, that means the HPV test (HPV detection by HP-II, PCR, HPV-DNA in situ hybridization and other methods) positive CIN1 patients as the research group. |
| C  Comparison or Control Group | Detailed reporting of the (control) group interventions is indispensable for the reader to fully understand the inclusion criteria of the included studies and may provide a heterogeneous source for the investigator.Comparative measures often lack adequate description. Clearly report interventions for comparison groups are very important. | The CIN1 patients with negative HPV testing were selected as the control group for this study. (Note: the diagnosis of CIN1 at the same time also the detection of HPV). |
| O  Outcome | The outcome of the assessed intervention (exposure) (eg, mortality, morbidity, symptoms, or improvement in quality of life) should be clearly defined as this is essential for the validity and applicability of the results of the systematic reviews / meta-analyzes. | Main Outcome Measures  Compared with HPV-negative,the risk of the CIN1 disease with HPV-positive progression, persistent and regression. |
|  |  | Secondary (Indirect) Outcome Indicators  Progression rate,persistent and regression rate of CIN1 lesions with HPV-positive or HPV-negative . |
| S  Research Design | The type of design to be included in the study should be reported.Some systematic reviews / Meta only included randomized trials, but some included a broader range of study designs (eg, randomized trials and some types of observational studies). There are also systematic reviews (eg, specific assessment of hazards) / meta-analyzes that incorporate a broad range of research design types from cohort studies to case reports. | This study selected control cohort studies (prospective / retrospective cohort studies). The control cohort study was one of the cohort studies and the control group was set up to compare with the exposure group. When exposed to a specific exposure group, an unexposed population as the control group, the two groups of relevant information to be collected at the same time. |

**Appendix 2: Full Search strategy**

| **Database and Search time** | **Search strategy** |
| --- | --- |
| PubMed | #1: "Squamous Intraepithelial Lesions of the Cervix"[MeSH] |
| 2016/03/29 | #2: (((low-grade squamous intraepithelial lesion) OR mild cervical dysplasia) OR CIN1) OR mild Cervical Intraepithelial Neoplasia |
|  | #3: #1 OR #2 |
|  | #4: "Human Papillomavirus DNA Tests"[MeSH] |
|  | #5: ((human papillomavirus detected) OR human papillomavirus test) OR human papillomavirus infection |
|  | #6: #4 OR #5 |
|  | #7: (Cohort Study) OR follow up |
|  | #8: #3 AND #6 AND #7 |
| Cochrane Library | #1: MeSH descriptor: [Squamous Intraepithelial Lesions of the Cervix] explode all trees |
| 2016/04/20 | #2: "low-grade squamous intraepithelial lesion" OR "mild cervical dysplasia" OR "CIN1" OR "mild Cervical Intraepithelial Neoplasia" |
|  | #3: #1 OR #2 |
|  | #4: MeSH descriptor: [Human Papillomavirus DNA Tests] explode all trees |
|  | #5: "human papillomavirus detected" OR "human papillomavirus test" OR "human papillomavirus infection" |
|  | #6: #4 OR #5 |
|  | #7: "Cohort Study" OR "follow up" |
|  | #8: #3 AND #6 AND #7 |
| EMBASE | #1: "Squamous Intraepithelial Lesions of the Cervix"/exp |
| 2016/05/06 | #2: (“low-grade squamous intraepithelial lesion” OR “mild cervical dysplasia” OR “CIN1” OR “mild Cervical Intraepithelial Neoplasia”).af. |
|  | #3: #1 OR #2 |
|  | #4: Human Papillomavirus DNA Tests /exp |
|  | #5: (“human papillomavirus detected” OR “human papillomavirus test” OR “human papillomavirus infection”).af. |
|  | #6: #4 OR #5 |
|  | #7: (“Cohort Study” OR “follow up”).af. |
|  | #8: #3 AND #6 AND #7 |
| CNKI | SU=(HPV+Papilloma virus)*(Cervical intraepithelial neoplasia grade Ⅰ+ CIN1 + mild cervical intraepithelial neoplasia+ LSIL + low-grade cervical squamous intraepithelial lesions) * (Natural History + Outcome + Prognosis + Progression + Regression + Persistence +Transition Probability + Follow-up) |
| 2016/03/29 |  |
| WANFANG | Abstract=("HPV" or "Human papilloma virus "or"papilloma virus ") and Abstract=("Cervical intraepithelial neoplasia grade Ⅰ" or "CIN1" or "mild cervical intraepithelial neoplasia" or "LSIL" or "low-grade cervical squamous intraepithelial lesions") and Abstract=("Natural History" or "Outcome" or "Prognosis" or "Progression" or "Regression" or "Persistence" or "Transition Probability" or "Follow-up") |
| 2016/03/29 |  |
| CBM | ("HPV"[Abstract] or "Human papilloma virus"[Abstract] or "papilloma virus"[Abstract]) and ("Cervical intraepithelial neoplasia grade Ⅰ"[Abstract] or "CIN1"[Abstract] or "mild cervical intraepithelial neoplasia"[Abstract] or "LSIL"[Abstract] or "low- grade cervical squamous intraepithelial lesions"[Abstract]) and ("Natural History"[Abstract] or "Outcome"[Abstract] or "Prognosis" [Abstract] or "Progression"[Abstract] or "Regression"[Abstract] or "Persistence"[Abstract] or "Transition Probability"[Abstract] or "Follow-up"[Abstract]) |
| 2016/04/22 |  |

**Appendix 3. Newcastle-Ottawa Quality Assessment Scale: Cohort Studied**

| **Studies** | **Study population selection** | | | | | | | | | | | | |
| --- | --- | --- | --- | --- | --- | --- | --- | --- | --- | --- | --- | --- | --- |
|  | Representativeness of the exposed cohort (1 point) | | | | Selection of the non exposed cohort (1 point) | | | Ascertainment of exposure (1 point) | | | | Demonstration that outcome of interest was not present at start of study (1 point) | |
|  | Truly representative of the average in the community* | Somewhat representative of the average in the community* | Selected group of users eg nurses, volunteers | No description of the derivation of the cohort | Drawn from the same community as the exposed cohort* | Drawn from a different source | No description of the derivation of the non exposed cohort | Secure record (eg surgical records)* | Structured interview* | Written self report | No description | Yes* | No |
| Sagasta (2016) |  | √ |  |  | √ |  |  | √ |  |  |  | √ |  |
| Veijalainen (2015) |  | √ |  |  | √ |  |  | √ |  |  |  | √ |  |
| He (2015) | √ |  |  |  | √ |  |  | √ |  |  |  | √ |  |
| Zhou (2015) |  | √ |  |  | √ |  |  | √ |  |  |  | √ |  |
| Mou (2014) |  | √ |  |  | √ |  |  | √ |  |  |  | √ |  |
| Siriaunkgul (2014) |  | √ |  |  | √ |  |  | √ |  |  |  | √ |  |
| Hu (2014) | √ |  |  |  | √ |  |  | √ |  |  |  | √ |  |
| Jiang (2013) |  | √ |  |  | √ |  |  | √ |  |  |  | √ |  |
| Waldstrøm (2013) |  | √ |  |  | √ |  |  | √ |  |  |  | √ |  |
| Katki (2013) |  | √ |  |  | √ |  |  | √ |  |  |  | √ |  |
| Byun (2013) |  | √ |  |  | √ |  |  | √ |  |  |  | √ |  |
| Liao (2013) |  | √ |  |  | √ |  |  | √ |  |  |  | √ |  |
| Li (2013) |  | √ |  |  | √ |  |  | √ |  |  |  | √ |  |
| Wang (2012) | √ |  |  |  | √ |  |  | √ |  |  |  | √ |  |
| Huang (2012) |  | √ |  |  | √ |  |  | √ |  |  |  | √ |  |
| Bowring (2012) |  | √ |  |  | √ |  |  | √ |  |  |  | √ |  |
| Jakobsson (2012) |  | √ |  |  | √ |  |  | √ |  |  |  | √ |  |
| Ozaki (2011) |  | √ |  |  | √ |  |  | √ |  |  |  | √ |  |
| Li (2011) |  | √ |  |  | √ |  |  | √ |  |  |  | √ |  |
| Gonzalez-Bosquet (2010) |  | √ |  |  | √ |  |  | √ |  |  |  | √ |  |
| Waldstrøm (2010) | √ |  |  |  | √ |  |  | √ |  |  |  | √ |  |
| Heider (2010) | √ |  |  |  | √ |  |  | √ |  |  |  | √ |  |
| Cotton (2010) |  | √ |  |  | √ |  |  | √ |  |  |  | √ |  |
| Thrall (2009) |  | √ |  |  | √ |  |  | √ |  |  |  | √ |  |
| Liao (2008) |  | √ |  |  | √ |  |  | √ |  |  |  | √ |  |
| Gong (2007) |  | √ |  |  | √ |  |  | √ |  |  |  | √ |  |
| Santos (2006) |  | √ |  |  | √ |  |  | √ |  |  |  | √ |  |
| Tarkkanen (2006) |  | √ |  |  | √ |  |  | √ |  |  |  | √ |  |
| SONG (2006) |  | √ |  |  | √ |  |  | √ |  |  |  | √ |  |
| Clavel (2005) |  | √ |  |  | √ |  |  | √ |  |  |  | √ |  |
| Massad (2004) |  | √ |  |  | √ |  |  | √ |  |  |  | √ |  |
| Alameda (2004) |  | √ |  |  | √ |  |  | √ |  |  |  | √ |  |
| Sastre-Garau (2004) |  | √ |  |  | √ |  |  | √ |  |  |  | √ |  |
| Schlecht (2003) |  | √ |  |  | √ |  |  | √ |  |  |  | √ |  |
| Denise (2001) | √ |  |  |  | √ |  |  | √ |  |  |  | √ |  |
| Matsuura(1997) |  | √ |  |  | √ |  |  | √ |  |  |  | √ |  |
| Kaufman (1997) |  | √ |  |  | √ |  |  | √ |  |  |  | √ |  |
| Campion (1986) |  |  |  | √ | √ |  |  | √ |  |  |  | √ |  |

(continue)

| **Studies** | **Comparability between groups** | | **Outcome measure** | | | | | | | | | | The total score |
| --- | --- | --- | --- | --- | --- | --- | --- | --- | --- | --- | --- | --- | --- |
|  | Comparability of cohorts on the basis of the design or analysis(2 points) | | Assessment of outcome(1 poin) | | | | Was follow-up long enough for outcomes to occur(1 point) | | Adequacy of follow up of cohorts  (1 point) | | | |  |
|  | Study controls for most important factor* | Study controls for any additional factor* | Independent blind assessment* | Record linkage* | Self report | No description | Yes（≥ 6 months）* | No | complete follow up* | subjects lost to follow up unlikely to introduce bias(select an adequate %) follow up, or description provided of those lost) * | Has follow up rate (select an adequate %)and no description of those lost | no statement |  |
| Sagasta (2016) | √ |  |  | √ |  |  | √ |  | √ |  |  |  | 8 |
| Veijalainen (2015) | √ |  |  | √ |  |  | √ |  |  | √ |  |  | 8 |
| He (2015) | √ | √ |  | √ |  |  | √ |  | √ |  |  |  | 9 |
| Zhou (2015) |  |  |  | √ |  |  | √ |  | √ |  |  |  | 7 |
| Mou (2014) | √ |  |  | √ |  |  | √ |  |  |  |  | √ | 7 |
| Siriaunkgul (2014) | √ |  |  | √ |  |  | √ |  |  | √ |  |  | 8 |
| Hu (2014) | √ | √ | √ |  |  |  | √ |  |  | √ |  |  | 9 |
| Jiang (2013) | √ |  |  | √ |  |  | √ |  | √ |  |  |  | 8 |
| Waldstrøm (2013) | √ |  |  | √ |  |  | √ |  | √ |  |  |  | 8 |
| Katki (2013) | √ |  |  | √ |  |  | √ |  | √ |  |  |  | 8 |
| Byun (2013) | √ |  |  | √ |  |  | √ |  | √ |  |  |  | 8 |
| Liao (2013) | √ |  |  | √ |  |  | √ |  | √ |  |  |  | 8 |
| Li (2013) |  |  |  | √ |  |  | √ |  | √ |  |  |  | 7 |
| Wang (2012) | √ | √ | √ |  |  |  | √ |  | √ |  |  |  | 9 |
| Huang (2012) |  |  |  | √ |  |  | √ |  | √ |  |  |  | 7 |
| Bowring (2012) | √ |  |  | √ |  |  | √ |  | √ |  |  |  | 8 |
| Jakobsson (2012) | √ |  | √ |  |  |  | √ |  |  | √ |  |  | 8 |
| Ozaki (2011) | √ |  |  | √ |  |  | √ |  |  |  | √ |  | 7 |
| Li (2011) | √ |  |  | √ |  |  | √ |  |  | √ |  |  | 8 |
| Gonzalez-Bosquet (2010) |  |  |  | √ |  |  | √ |  | √ |  |  |  | 7 |
| Waldstrøm (2010) | √ |  |  | √ |  |  | √ |  |  | √ |  |  | 8 |
| Heider (2010) | √ | √ |  | √ |  |  | √ |  | √ |  |  |  | 9 |
| Cotton (2010) | √ |  |  | √ |  |  | √ |  | √ |  |  |  | 8 |
| Thrall (2009) | √ | √ |  | √ |  |  | √ |  | √ |  |  |  | 9 |
| Liao (2008) | √ |  |  | √ |  |  | √ |  |  |  | √ |  | 7 |
| Gong (2007) | √ |  |  | √ |  |  | √ |  |  |  |  | √ | 7 |
| Santos (2006) | √ |  |  | √ |  |  | √ |  |  | √ |  |  | 8 |
| Tarkkanen (2006) |  |  |  | √ |  |  | √ |  | √ |  |  |  | 7 |
| SONG (2006) | √ |  |  | √ |  |  | √ |  |  | √ |  |  | 8 |
| Clavel (2005) | √ |  |  | √ |  |  | √ |  | √ |  |  |  | 8 |
| Massad (2004) | √ |  |  | √ |  |  | √ |  |  | √ |  |  | 8 |
| Alameda (2004) |  |  |  | √ |  |  | √ |  | √ |  |  |  | 7 |
| Sastre-Garau (2004) | √ |  |  | √ |  |  | √ |  | √ |  |  |  | 8 |
| Schlecht (2003) | √ |  |  | √ |  |  | √ |  | √ |  |  |  | 8 |
| Denise (2001) | √ |  | √ |  |  |  | √ |  |  | √ |  |  | 8 |
| Matsuura(1997) | √ |  |  | √ |  |  | √ |  |  | √ |  |  | 8 |
| Kaufman (1997) | √ |  |  | √ |  |  | √ |  |  |  |  | √ | 7 |
| Campion (1986) | √ |  |  | √ |  |  | √ |  | √ |  |  |  | 7 |

Note: *, representing the option to score

**Appendix 4:** Results of the subgroup analyses of the effects of CIN1 disease outcomes of relative factors. RR, relative risk; HR-HPV, High-risk HPV; LR-HPV, Low-risk HPV.


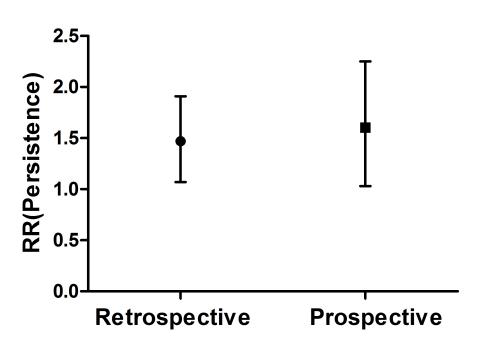

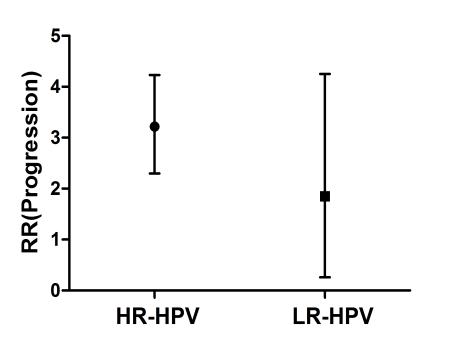

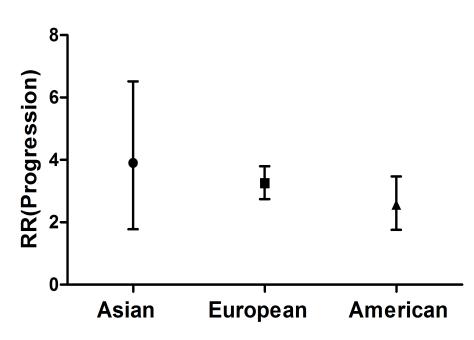

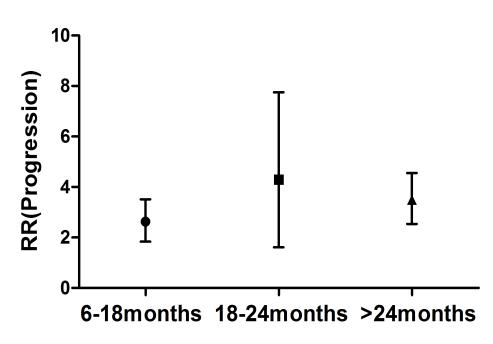

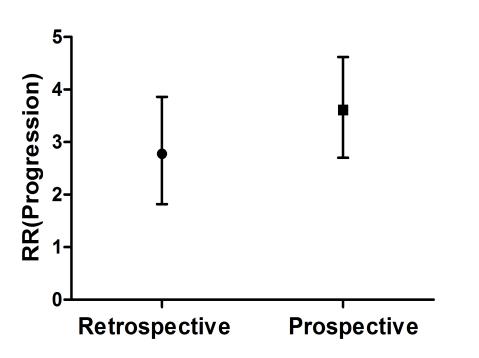

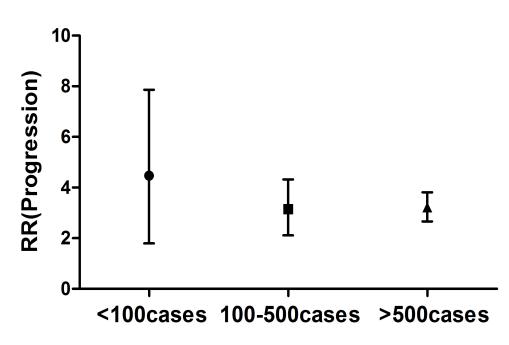

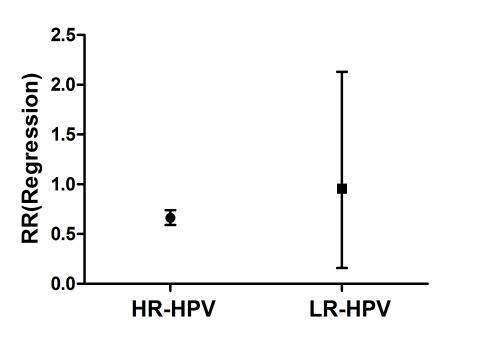

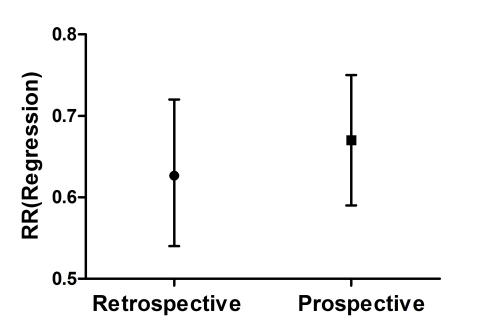

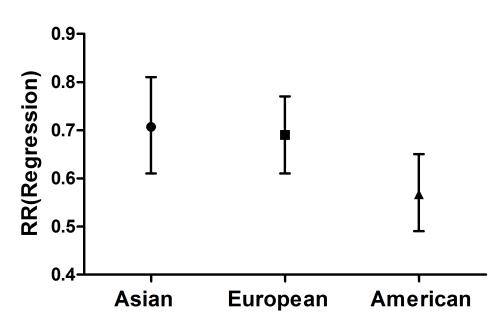

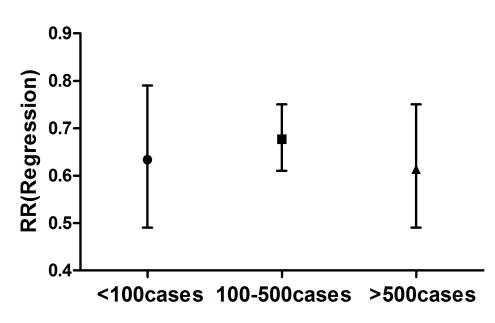

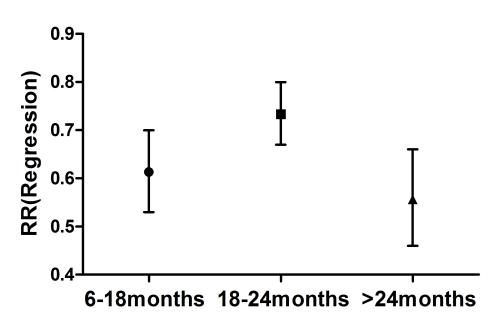

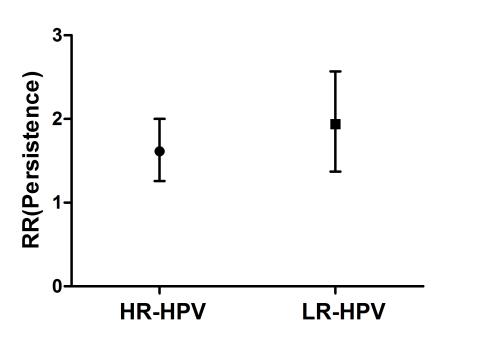

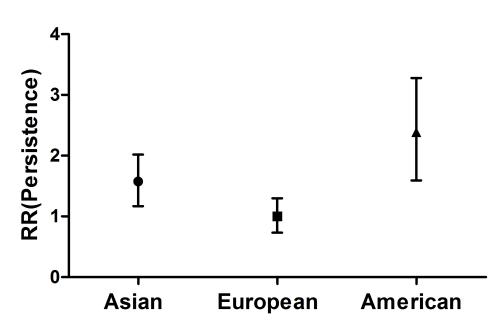

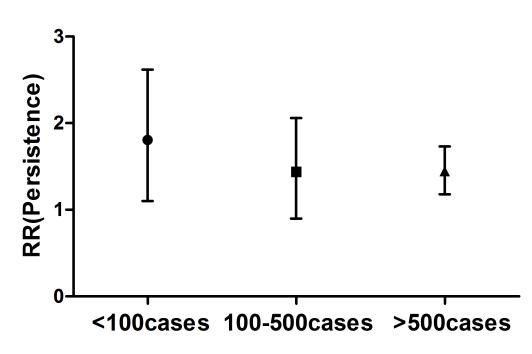

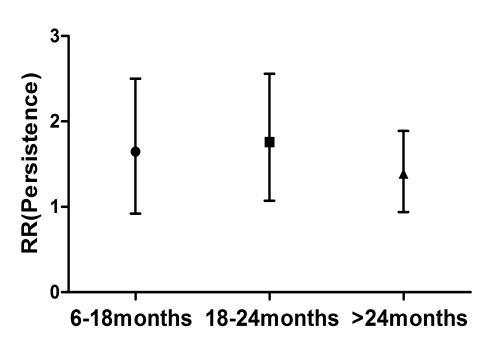


**Appendix 5:**Sensitivity analysis for the pregression(A), persistence(B) and regression(C) of CIN1 disease. CI, confidence interval.

A B


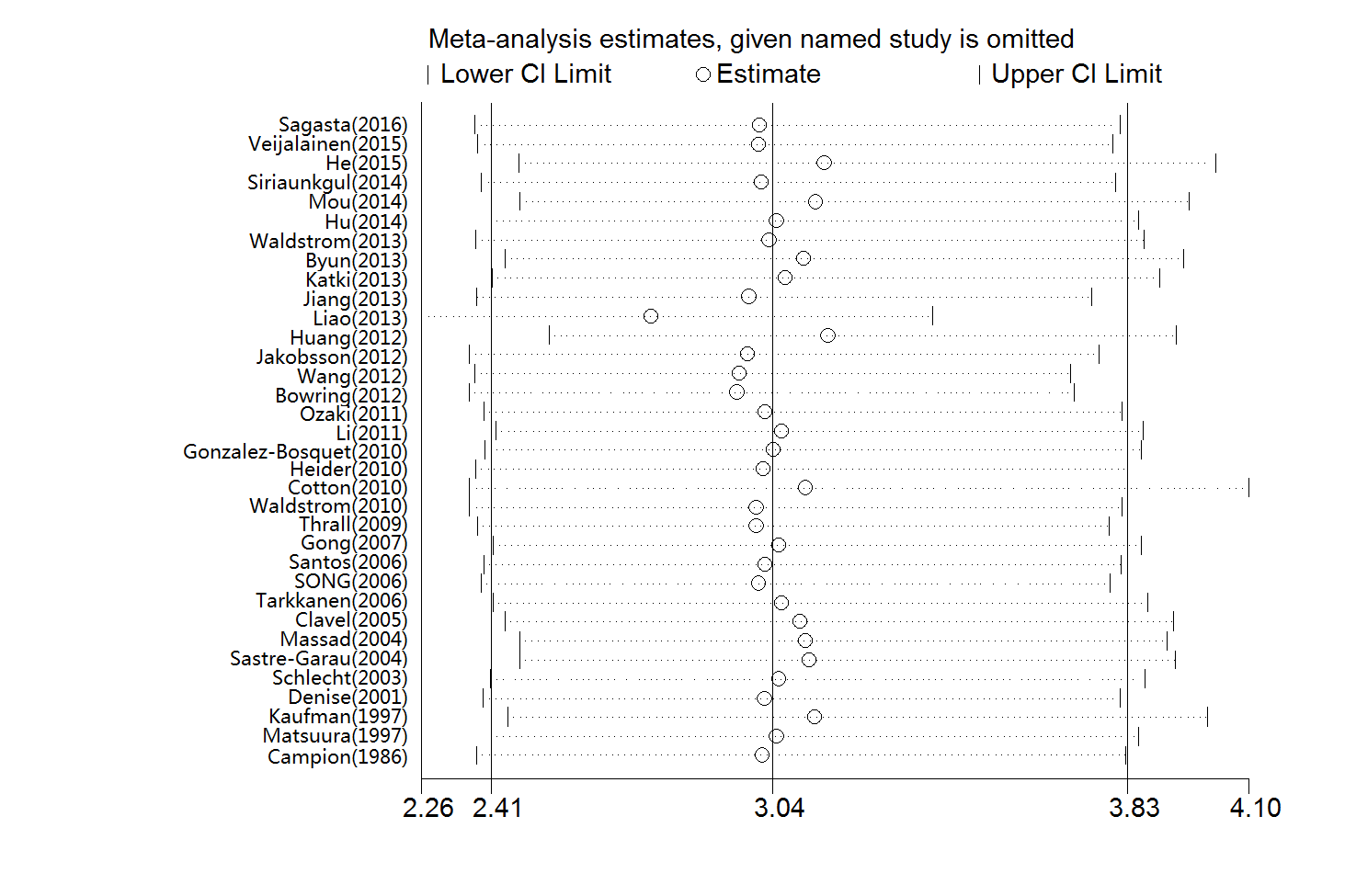

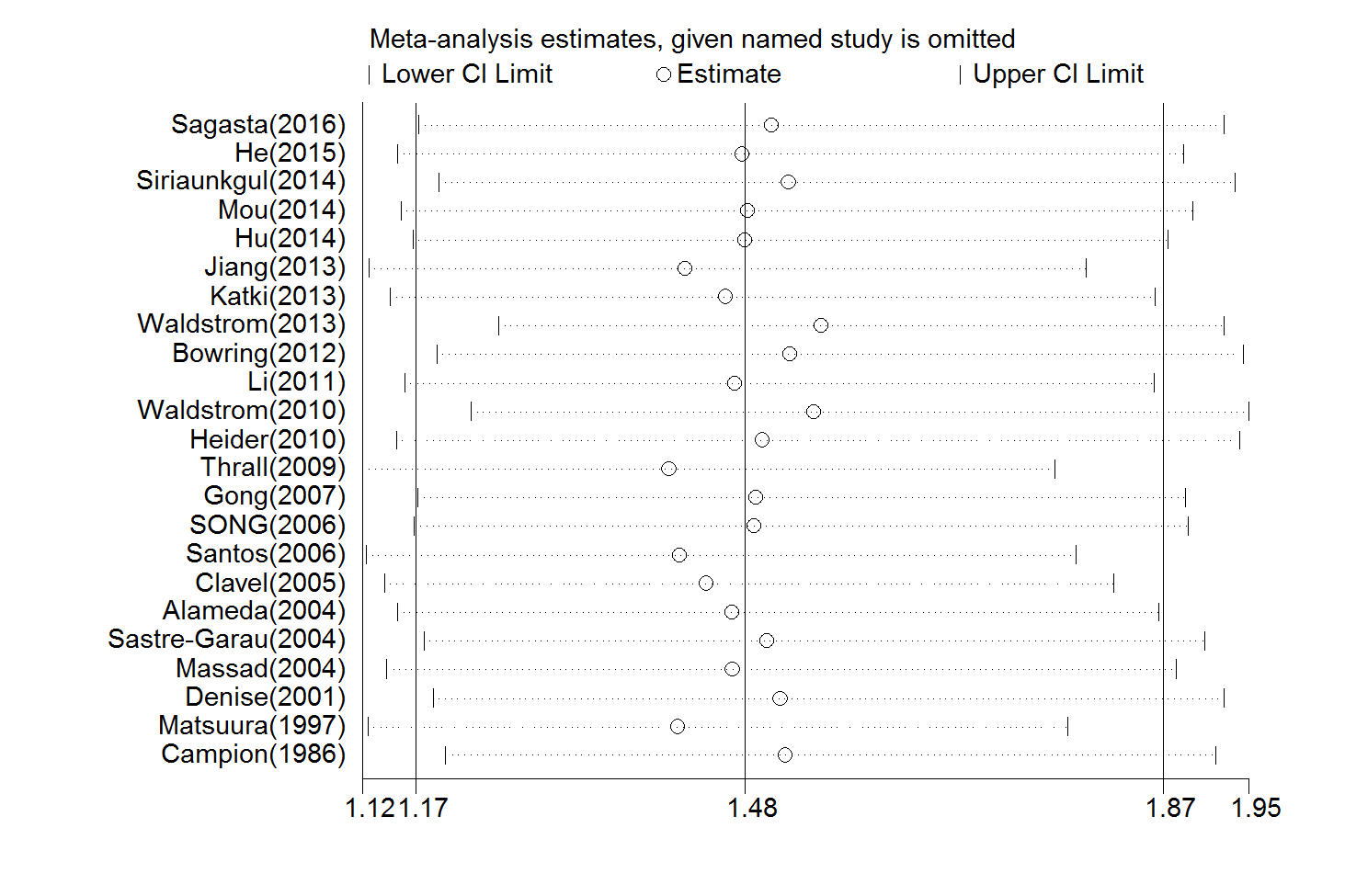

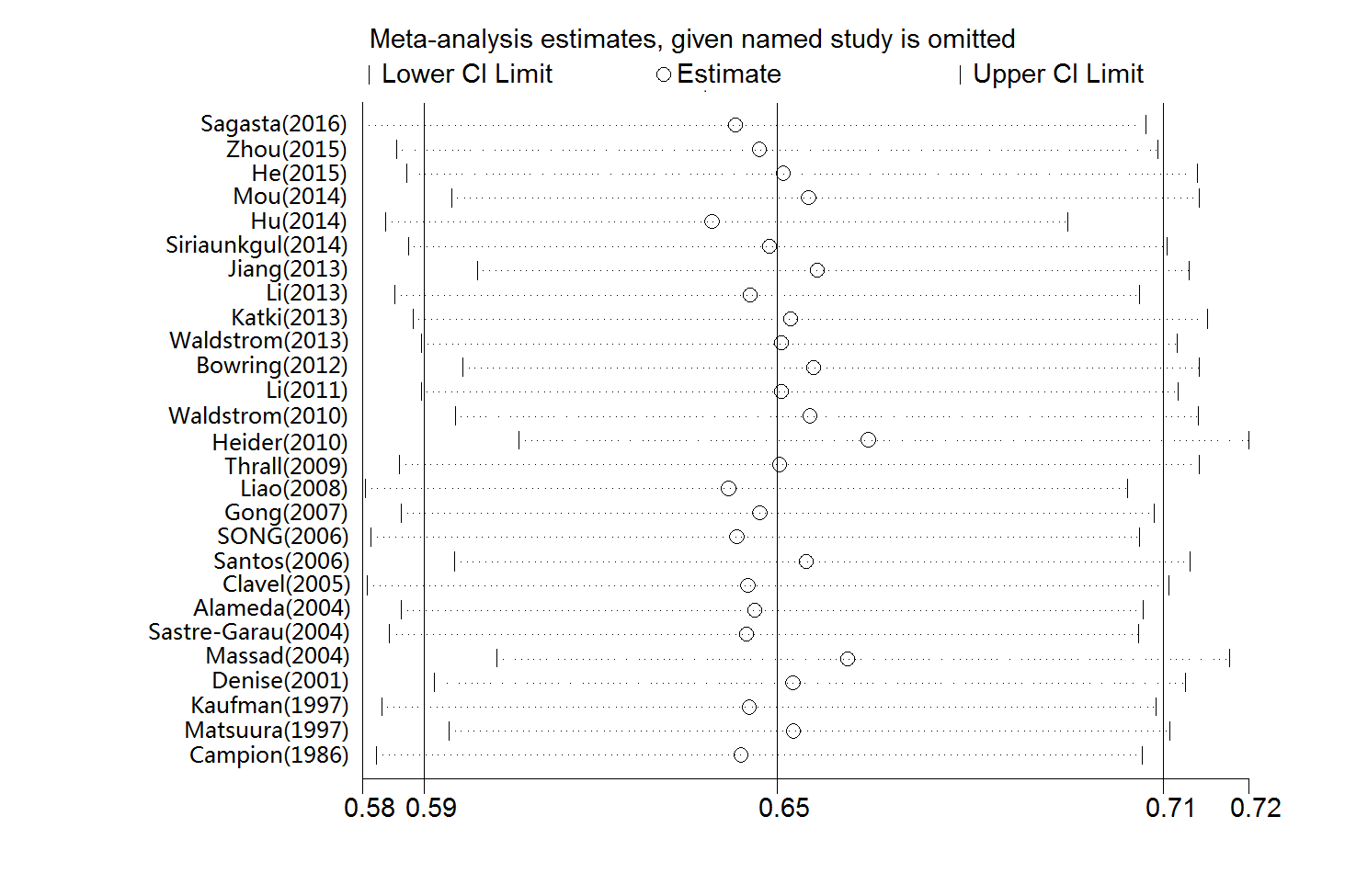


C
